# Stability testing of resveratrol and viniferin obtained from *Vitis vinifera* L. by various extraction methods considering the industrial viewpoint

**DOI:** 10.1038/s41598-020-62603-w

**Published:** 2020-03-27

**Authors:** Ema Kosović, Martin Topiař, Petra Cuřínová, Marie Sajfrtová

**Affiliations:** 1 University of Chemistry and Technology in Prague, Technická 5, Prague, 6, 16 628 Czech Republic; 2 Institute of Chemical Process Fundamentals of CAS v.v.i., Rozvojová 135, Prague, 6, 16502 Czech Republic

**Keywords:** Abiotic, Light stress, Heat, Light responses

## Abstract

Solid by-products generated in the winemaking process, can comprise valuable bioactive substances such as resveratrol and viniferin, which can be used in whole range of sectors including medicine, pharmacy, cosmetic industry etc. The changes in content of those stilbenes in extracts obtained by maceration and Soxhlet extraction were monitored using newly modified and validated high-performance liquid chromatography-mass spectrometry method which was proved to be accurate, reproducible, and efficient for their determination. The yields of individual bioactive compounds isolated from winery by-products are crucially dependent on the conditions of used extraction techniques. From this point of view, stability testing including light exposure, elevated temperature, and storage for longer time periods in the solution, represents the basis for optimizing conditions of extraction methods of resveratrol and *trans*-ε-viniferin. High temperature is beneficial for better release of thermally more stable stilbenes such as *trans*-resveratrol and *trans*-ε-viniferin but its application for prolonged time periods can be destructive. Light stress conditions cause the formation of otherwise unavailable *cis*-ε-viniferin by dimerization and photoisomerization of *trans-* stilbenes.

## Introduction

Wine industry produces around 20% of waste mainly composed by grape pomace and grape cane^1^. Grape pomace is a mixture of seeds and husks containing oil, anthocyanins, and stilbenes^2^, but grape cane was more investigated as a source of high-value phytochemicals with very diverse applications. Resveratrol and viniferin as the most common stilbene derivatives in grape cane, also known as phytoalexins, are important in food production and also can be very useful in various branches of science thanks to their potential antioxidant activity^1,3^. Several studies have confirmed their cardioprotective^4,5^, neuroprotective^6–8^, chemopreventive^9,10^, antimutagenic^11^, and antiaging effects^12^, however, the *in vivo* applications are still rare due to their limited stability and low bioavailability^13,14^.

Over the years, scientists have studied different varieties of grapevine, wine and grape juices using extraction methods under various conditions and solvents in order to find a methodology leading to extracts with maximum yield of bioactive compounds^15,16^. In 2006, Pussa *et al*.^17^ made a survey of grapevine stem polyphenols as an additional source for these highly valuable bioactive compounds. From that point on, research on the grapevine variety using extraction methods under different conditions in order to find a methodology leading to extracts with a maximum yield of bioactive compounds, is a topic of great interest. Rayne *et al*.^18^ started with finding the best solvent for stilbenes extraction. The quantitative extraction of phenolic compounds from grape cane was compared using a wide range of protic and aprotic solvents systems, among which the best results were achieved using a mixture of ethanol and water. Although the order of decreasing capacity to extract soluble materials was ethanol > methanol > water, in a major number of studies methanol was used as the most selective option for extracting of phenolic compounds, including stilbenes^19^. This finding was also confirmed by Soural *et al*.^20^ who studied the effectiveness of several extractive techniques for obtaining *trans*-resveratrol, *trans*-ε-viniferin, and R2-viniferin, where the highest amounts of all stilbenes were obtained using accelerated solvent extraction in methanol. Although it provides the best results due to its physical properties, methanol is not free of restrictive governmental regulations in terms of industrial production. Karacabey *et al*.^21^ continued with observation of the concentration change of stilbenes in dependence on temperature for short time intervals, for maximum of 60 minutes using pressurized low-polarity water extractor. They concluded that the effect of temperature is significant for those compounds especially in case of *trans*-resveratrol where it came to 39% loss at 150 °C compared with 95 °C. Moreover, according to Tříska *et al*.^22^ the content of stilbenes can differ among the same varieties harvested in different years and location.

Observation of pH, thermal stability, and solubility of resveratrol by Župančič *et al*.^13^ gave an important overview about *in vitro* properties of resveratrol. While several other publications provided further data concerning the chemical, biochemical, and physical properties of this stilbene^13,23–26^, to the best of our knowledge, no other study has focused on influence of various environmental factors such as temperature, light, and stability tests duration on extraction methods. The aim of this study was, therefore, implementation of experimental design, including stability tests, to determine the best extraction conditions that maximize the selectivity and extraction yield of each of the biologically most important stilbenes, *trans*-ε-viniferin, *cis*-ε-viniferin, and *trans-*resveratrol using industrially acceptable solvent - isopropyl alcohol.

## Results and Discussion

### HPLC-DAD/MS method

**Figure 1:**
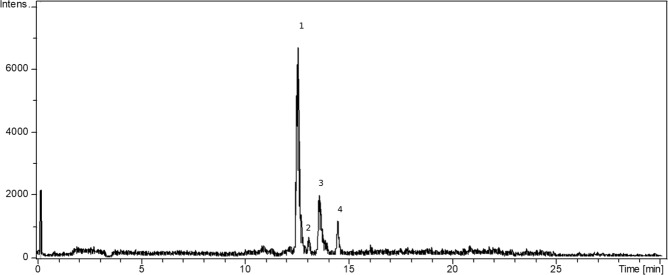
Chromatogram of all three stilbenes in full-scan (negative mode with electrospray ionization). 1: *trans*-resveratrol; 2: *cis*-ε-viniferin; 3: *trans*-ε-viniferin; 4: r2-viniferin.

Newly modified reversed-phase HPLC-DAD/MS method^27^ was used for assay of *trans*-resveratrol and *trans*-ε-viniferin. The new method uses steeper gradient profile and slower flow rate with a difference also in ESI ionization source parameters. Injected samples with a mass concentration around 5 mg/mL were monitored at 306 nm and chromatographic peaks were identified by comparing retention times UV and MS spectra of the samples with those of the standard compounds. Although all three compounds were visible in UV at 306 nm, high sensitive MS detection was used to determine their content. Retention times of individual monitored compounds were 12.5 min, 13.7 min, and 14.3 min for *trans*-resveratrol, *cis*-ε-viniferin, and *trans*-ε-viniferin, respectively (Fig. 1). The NMR method mentioned in subdivision 2.7 was used to distinguish between *cis* and *trans* form of ε-viniferin.

Since MS provides both, confirmation of peaks identity and more sensitive detection than UV, calibration with six standard dilutions of *trans*-resveratrol and five of *cis* and *trans*-ε-viniferin was performed only based on MS spectra. The linear relationship between the peak area and the concentration was reached over the selected range of mass concentrations 0.042–0.653 mg/L for *trans*-resveratrol and 0.05–0.35 mg/L for *cis* and *trans*-ε-viniferin with appreciable recovery rates (98.52% and 101.20%) of quality controls at improved detection and quantitation limits.

All experimental data were in the range of the acceptability for accuracy, precision, and reproducibility and the modified method gives reproducible results with standard deviation of 0.001–0.095 for 10 replicate injection^28,29^. Sensitivity presented using LOD and LOQ parameters showed, that lowest detected amount of *trans*-resveratrol was 9.03 µg/mL, while *cis* and *trans*-ε-viniferin showed higher detection limit of 13.68 µg/mL and 19.34 µg/mL, respectively. The lowest quantitation limit was determined at 48 µg/mL for *trans*-resveratrol and 68.7 µg/mL for both isomers of viniferin.

Determination of concentration of those three stilbenes in extracts by this method proved to be accurate using a simple procedure, without sample pre-treatment other than dilution.

### Extraction efficiency

**Grape pomace**, containing dried solid remains of skins, seeds, stems and pulp, was extracted by Soxhlet extraction (SOX) and maceration (MAC) in isopropyl alcohol (IPA). According to standard Folin-Ciocalteu test^30^, the content of phenolic compounds in the extract was found to be very low using SOX in duration for 3 hours. To improve these results, extraction time was prolonged to 7 hours, when the extraction efficiency of this method increased to about 12% and extract contained almost 26 mg GAE/g of phenolic compounds. Extending the extraction time as a method of improving the results has been mentioned in several publications^31,32^, but Naczk *et al*.^33^ showed that longer period not always enhance the extraction efficiency. Thus, extraction efficiency of maceration in duration of 24 hours was about 7% obtaining 24.42 mg GAE/g of phenolic compounds. In conclusion, SOX proved to be faster and more efficient method in terms of obtained yields of phenolic compounds. This was also confirmed by statistically significant difference between those two methods in terms of velocity-dependent extraction efficiency, using two way ANOVA (α = 0.01, n = 12, p = 5.76 × 10^−5^) with post hoc Tukey test.

Nevertheless, HPLC-MS analysis of obtained samples did not show the presence of resveratrol and viniferin isomers. Quercetin, chlorogenic acid, pelargonin, galocathol, and sinapaldehyde were found instead.

Milled and dried **grape canes** were extracted also by SOX, light exposed MAC and MAC protected from light. For reason of result comparison and finding of the difference, MAC protected from light was repeated at 45 °C. Folin-Ciocalteu test proved that phenolic compounds in grape cane were best extracted by maceration performed for 3 days on light. Extraction efficiency was 1.5% obtaining 17.7 mg GAE/g of phenolic compounds. To accelerate the whole procedure, ultrasound assisted extraction at elevated temperature (40 °C) was used, where the same amount of phenolic compounds (17.7 mg GAE/g) is extracted within 10 min with same extraction efficiency around 1.3%. Based on literature, this extraction technique is largely used for extraction of phenolic compounds from plant materials thanks to simple equipment, reduced extraction time and solvent consumption^34,35^. Statistically significant difference was confirmed between maceration and ultrasound extraction in terms of velocity-dependent extraction efficiency, using two-way ANOVA (α = 0.01, n = 12, p = p = 9.68 × 10^−4^) with post hoc Tukey test.

Unlike grape pomace extracts, the extracts from grape canes contained among the phenolic compounds also the target stilbenes, *trans*-resveratrol, *cis* and *trans*-ε-viniferin, in amounts and mutual ratio dependent on the conditions applied on used extraction technique.

**Figure 2:**
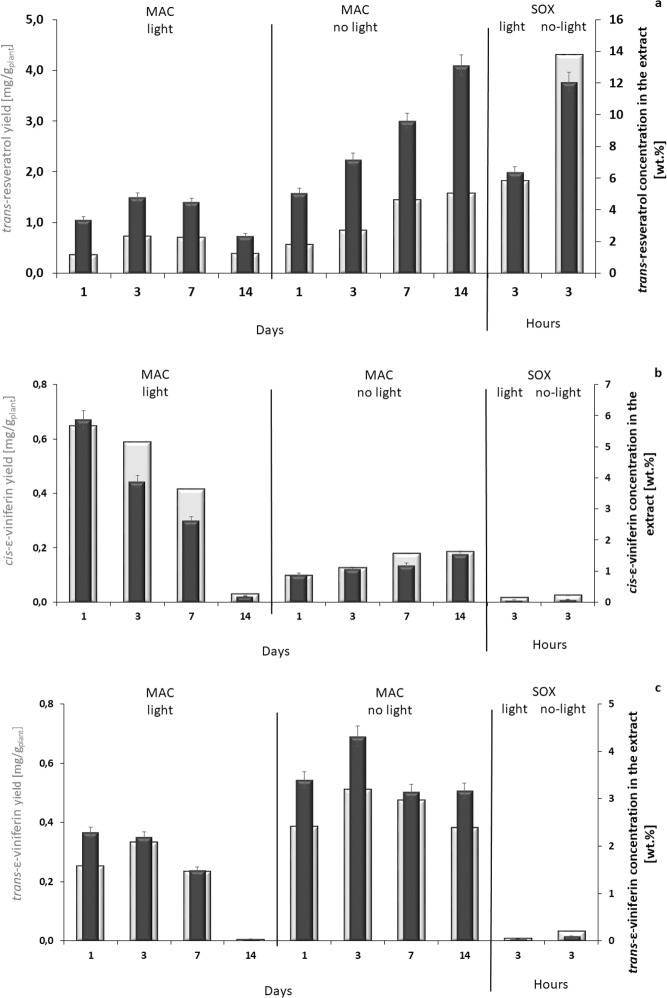
(**a**) *trans*-resveratrol, (**b**) *cis*-ε-viniferin, and (**c**) *trans*-ε-viniferin - Comparison of maceration (MAC) carried out for 1, 3, 7, and 14 days and Soxhlet extraction (SOX) for 3 hours, both in light and dark with respect to the yield of desirable components (white column), mg/g_plant_ and their concentration in extracts (black column), wt.% (based on total mass of extract) with 95% confidence intervals.

The component yield and component concentration in the extract of *trans*-resveratrol, *cis*, and *trans*-ε-viniferin are summarized in Fig. 2. The highest yield of resveratrol with the value of 1.83 mg/g containing 13.12% of *trans*-resveratrol was obtained by maceration in dark at laboratory temperature (Fig. 2a). The longer time of extraction in dark provides higher concentration of *trans*-resveratrol without significant decomposition of this compound or substantial increase of extracted ballast. On the other hand, maceration carried out exposed to ambient light gave lower concentration of *trans-*resveratrol (4.8%) which even decreased over time to 2.3%. Improvement of maceration efficiency for this stilbene is possible using an elevated temperature (45 °C), resulting in an increased efficiency of about 5%. But after three days at these conditions the decomposition of target compound prevails. Decreasing of content of stilbenes exposed to light can be explained through degradation, derivatisation, or structural rearrangements^36^.

The extraction performed using Soxhlet method follows the same principles; much higher concentration of *trans*-resveratrol (12.07%) is obtained in dark with yield about 4.03 mg/g.

Extraction of *trans*-ε-viniferin (Fig. 2b,c), showed that maceration technique provided approximately 0.65 mg/g of *cis*-ε-viniferin versus 0.03 mg/g of the same compound obtained by Soxhlet. The highest concentration of *cis-*form containing 5.86% was found in the extracts macerated for the shortest time period, exposed to light, while the longer periods of time in the solution led to excessive degradation of this compound. This phenomenon is especially pronounced with the use of elevated temperature. Extraction of *trans*-ε-viniferin using maceration at shorter time periods with protection against light provided once again higher yield with value of 0.511 mg/g containing 4.32% of *trans*-ε-viniferin concentration in the extract (Fig. 2c).

### Stability of extracted components

**Figure 3:**
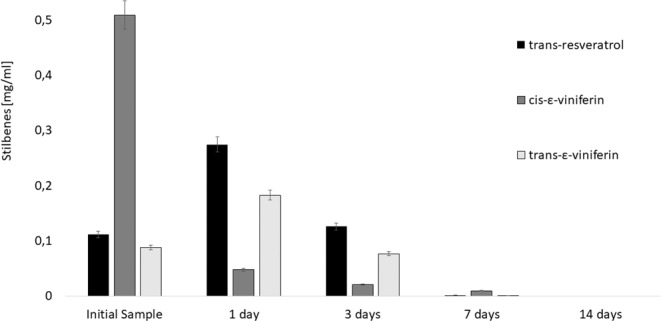
Influence of high temperatures on stilbenes mass concentration (mg/mL) in extracts protected from light (95% confidence intervals).

To understand the conditions of extraction and obtained concentration of individual compounds, we performed the decomposition studies with extracts containing high amount of compounds of interest. **Thermal stress results** shown in Fig. 3 lead to the conclusion, that content of *trans-*resveratrol and *trans*-ε-viniferin slightly increases by storage at elevated temperature of 45 °C and protected from light, before total degradation.

In the plant material, and surprisingly even in the extracts, a large portion of these stilbenes still remains bound to other molecular structures, such as saccharides. An explanation for this possible mechanism, described in the Methods in polyphenol analysis^37^, is that heat causes cell wall permeability, increases the solubility and diffusion coefficients of the compounds to be extracted, and reduces the viscosity of the solvent thus facilitating its passage through the solid substrate causing a doubling their weight.

The thermal stability of *cis*-ε-viniferin was very low with rapid decomposition. Almost nine times lower concentration of this compound was found after one day of storage at elevated temperature. Consideration should be given to the low thermal stability of stilbenes, especially in the case of *cis*-ε-viniferin, and heating longer than one day should be avoided.

**Figure 4:**
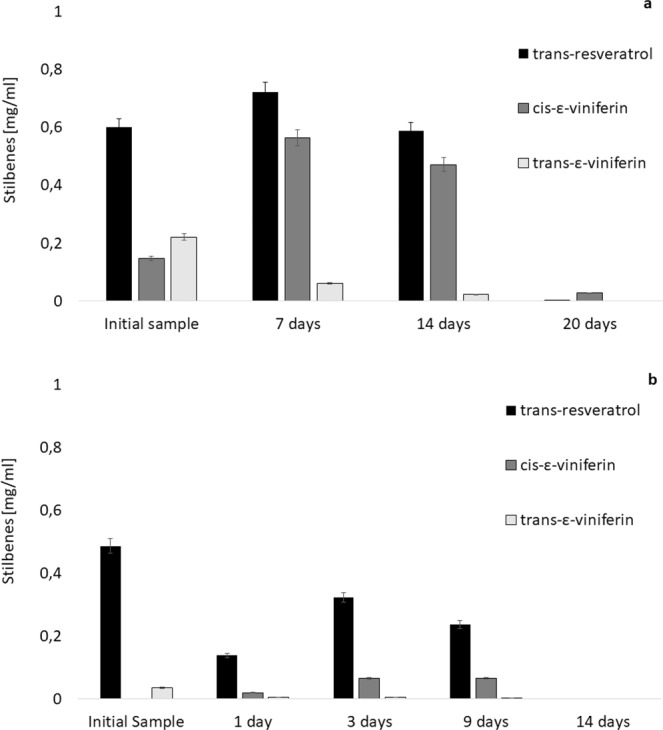
Influence of sun-light (**a**) and UV-light (**b**) exposure on stilbenes mass concentrations (mg/mL) with 95% confidence intervals.

**Exposure to light** can result in permanent damage of light-sensitive compounds. Therefore, the samples solubilized in IPA containing high amounts of stilbenes were exposed to light at room temperature for the period of 20 days (Fig. 4a). Under these conditions of storage the concentration of *trans*-resveratrol and *trans*-ε-viniferin in samples slowly decreased until almost complete decomposition (20 days). Nevertheless, in the case of *cis*-ε-viniferin, the concentration after 7 days of light exposure increased by 300%.

**Time stability** was observed in all performed tests. Longer standing of extracts in IPA solution proved to be devastating for all the stilbenes. On the other hand, as the decomposition, especially at lower temperatures, is not very fast, the storage of the extracts in solution is possible and sometimes favorable. As additional quantities of *trans-*resveratrol and *trans*-ε-viniferin can be released from the plant matrices by the solvent even in the extracts, the longer storage can increase concentration of these stilbenes in solution (at ambient temperature, in dark). By storage of extract solutions exposed to light, the concentration of *cis*-ε-viniferin can be increased with optimum time of one day (ambient temperature).

### *Cis*-ε-viniferin formation

Exposure of real extracts to light leads to an increase of *cis*-ε-viniferin concentration of 300%, due to its light induced formation from both, *trans*-ε-viniferin and *trans*-resveratrol. These findings based on HPLC-MS were supported by measurement of NMR spectra to irrevocably confirm the *cis*-ε-viniferin formation. This phenomenon was also described previously by Keylor *et al*.^38^ and Szewczuk *et al*.^39^ together with the possible pathway of *cis*-ε-viniferin formation in light-induced dimerization of resveratrol. However, the literature refers also on possible photo-isomerization of *trans-*ε-viniferin to *cis*-form^23^. To provide additional evidence, confirmatory study including UV-light was carried out (Fig. 4b). After light exposure, the initial *cis*-ε-viniferin-free sample exhibited *cis-*ε-viniferin formation accompanied by decrease in concentrations of both previously present stilbenes, resveratrol and *trans-*ε-viniferin. The distinction between sunlight and UV-light was in acceleration of light-induced chemical processes by UV irradiation. Final result of tests performed on sun-light and UV-light for a maximum time interval of 20 days and 14 days, respectively was the decomposition of all the stilbenes.

Stability tests were performed on 7 samples, with no statistically significant differences between them. Statistical differences were observed between compounds and stability conditions within 7 days of exposition to heat and 14 days after light exposure. Calculations performed using one-way ANOVA, are summarized in Table 1.

**Table 1:** Tukey’s honest significance test in mg/mL for different conditions (one-way ANOVA: *significant = p < 0.01, n.s. = not significant, n = 7.

*Conditions*	*trans-resveratrol*	*cis-ε-viniferin*	*trans-ε-viniferin*
*Light*	*p = 5.01 × 10^−4^	*p = 2.8 × 10^−7^	*p = 4.4 × 10^−5^
*Dark*	*n.s*.	*n.s*.	*n.s*.
*45 °C*	*p = 6.9 × 10^−5^	*p = 9.96 × 10^−8^	*p = 4.5 × 10^−7^

Values are means calculated from three measurements).

## Materials and Methods

### Chemicals and reagents

**Figure 5:**
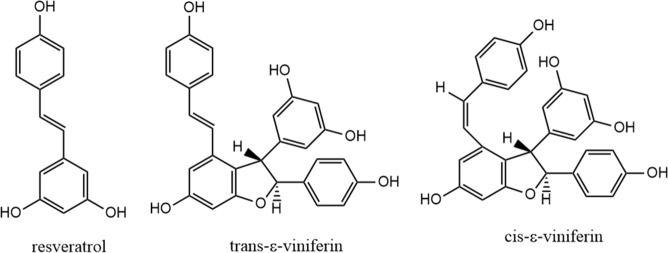
Structure of bioactive compounds extracted from wine waste.

The *trans*-resveratrol standard (99%) along with commercialized standard of *trans*-ε-viniferin (95%) (Fig. 5) were purchased from Sigma-Aldrich (Prague, Czech Republic), while the *cis*-ε-viniferin was generated from *trans*-ε-viniferin standard by UV exposition (Spectroline, CV-10, New York, USA). All compounds were measured as solutions in isopropyl alcohol (Lach-Ner, Prague, Czech Republic, gradient grade) which was also used as extraction solvent along with ethanol (Lach-Ner, Prague, Czech Republic, gradient grade). Other solvents for HPLC were methanol (Lach-Ner, Prague, Czech Republic, HPLC gradient grade), acetic acid (Lach-Ner, Prague, Czech Republic, 99%), and ultrapure water. Gallic acid (Sigma-Aldrich, Prague, Czech Republic, ACS reagent), Folin-Ciocalteu reagent (Sigma-Aldrich, Prague, Czech Republic, 2 M with respect to acid), sodium carbonate (Lachema, Brno, Czech Republic, 98%), and ultrapure water (Ultrapur Watrex system, Prague, Czech Republic) were used for determination of total content of phenolic compounds in extracts.

### Plant material

Samples of *Vitis vinifera L*. cv. Cabernet Sauvignon grown in Hustopeče (GPS: 48°56′27″N, 16°44′15″E), a town of Břeclav District in the South Moravian Region were analyzed. After pruning (February 2017) the **grape cane** material was spread on grids and dried at room temperature. Average water content in sample after drying was 8.5%. Chipping and grinding, followed by milling (heavy duty cutting mill, Retsch SM 200) shredded the material to approximate size of 1 mm. **Grape pomace** was dried also at room temperature in the same way as grape cane, and stripped from seeds while the rest (peels, thimbles) was milled (Retsch SM 200) to approximate size of 2 mm. Average water content in grape pomace sample after drying was 10.4%. Prepared material was stored at room temperature, in airtight containers without access to light for no longer than 1 month.

### Extraction

Various organic solvents can be used for extraction and analysis of stilbenes. In several studies, grapevine cane extracts were obtained using methanol or ethanol, which proved to be better extraction solvents than isopropyl alcohol. Nevertheless, from an industrial point of view, lower toxicity, flammability, and no restrictive government regulations led to employing isopropyl alcohol as the extraction solvent.

Maceration at laboratory temperature with and without exposure of extracted material to light and Soxhlet extraction under atmospheric pressure, used for extraction of plant extracts from grape pomace and cane, were compared in terms of extract yield and its chemical composition. Although, Vergara *et al*.^40^ published that 95% of total stilbenes recovered after four consecutive extractions, only one extraction was sufficient to compare the extraction efficiency of different techniques.

### Maceration

Grape pomace and grape cane extracts had the same preparation procedure. Plant material (20 g) was placed in Erlenmeyer flask filled with 200 ml of isopropyl alcohol. The sealed flask was put onto laboratory orbital shaker (GFL 3005) at room temperature. The rotation speed was set to 200 rotations per minute. Extraction time varied from 1 day (M1L) to 14 days on light (M14L). Carrying out the same experiment but without exposure to light (M1D-M14D), required an Erlenmeyer flask covered with aluminum foil.

### Soxhlet extraction

Plant material (12.5 g) was extracted with 200 ml of isopropyl alcohol in Soxhlet apparatus for 3 hours. The solvent was removed from the extract using a rotary vacuum evaporator at 30 °C (Rotavapor R-215, BUCHI, Switzerland). To perform the experiment in the dark, aluminum foil was used to cover the apparatus.

### Preparation of analytical samples

Forty five analytical samples were prepared by diluting of 25–30 mg of extract in 5 ml of isopropyl alcohol and measured in triplets using HPLC-MS. Seven samples of grape cane were subjected to stability testing.

As the stability tests include testing of stability-indicating attributes, such as temperature, light, and time stability (the end-of-day period at which complete degradation occurs), in this experiment, an attempt was made to change these parameters to observe their influence on the stilbene concentration in given extracts. The *cis*-ε-viniferin-rich sample, obtained by maceration on light for 24 hours was subsequently stored in dark at 45 °C, tested in thermal stability and measured in precise time intervals (1, 3, 7, and 14 days) by HPLC-MS. Photostability tests, had the same sampling schedule with extra sampling at longer exposition period of 20 days and were run at ambient temperature and at 0 °C. Initial samples, obtained by maceration in dark for 14 days and Soxhlet extraction in dark comprising a high concentration of *trans*-resveratrol, were exposed to day-light and UV light, respectively. For time based decomposition, a series of samples stored in dark at ambient temperature was measured.

All analytical samples were measured also for determination and quantification of phenolic compounds using Folin-Ciocalteu method, which presents colorimetric *in vitro* assay of (poly)-phenolic compounds^30^.

### Preparation of standard solutions

An alcoholic stock solution (isopropyl alcohol) of 0.4 mg/mL *trans*-resveratrol and 1 mg/mL of *cis* and *trans*-ε-viniferin, was prepared and stored at 4 °C. From stock solution, a set of 6 dilutions was prepared using 99% isopropyl alcohol. The set was used for generation of calibration curve for monitored compounds.

### HPLC-MS procedure

The experiment was carried out using a Dionex Ultimate 3000 HPLC system (Thermo Scientific, Waltham, Massachusetts, USA) equipped with diode array detector and Synergi Polar-RP 80 A column (150 mm ×4.6 mm I.D.; particle size 4 µm; Phenomenex, Torrance, CA, USA) with water-methanol-acetic acid mobile phase. Mobile phase consisted of a methanol/water/acetic acid (20:80:1, v/v) mixture as solvent A and methanol/water/acetic acid mixture (90:10:1, v/v) as solvent B at a flow rate of 0.5 mL.min^−1^ and column temperature of 25 °C. The multistep gradient had following profile: Pre-run 5 min 100% A, 0–10 min from 0% to 100% B, 100% B was maintained from 10 to 25 min and 25–30 min back from 100% to 0% B.

Injection volume was 5 µl. Detection was performed using DAD detector (Thermo Scientific, USA) at 306 nm wavelength.

For identity confirmation of *trans-*resveratrol and viniferins was used mass micrOTOF-Q III spectrometer (Bruker Daltonik, GmbH, Bremen, Germany) coupled to HPLC. The mass spectrometer was operated using electrospray ionization (ESI) source in negative mode with nitrogen as nebulizer (1.6 bar) and as drying gas (180 °C/8 L/min). All measurements by MS were performed with following parameters: the capillary voltage −2500 V, the end plate offset 500 V and the collision cell RF 350 Vpp. The mass range for the scans of MS spectra was *m/z* 80–1550 with low mass of 100 Da.

Collection of chromatographic and mass spectrometric data was carried out using HyStar 3.2 software (Bruker Daltonik, GmbH, Bremen, Germany) and for data processing Compass for otofSeries 1.5 Data Analysis 4.1. (Bruker Daltonik, GmbH, Bremen, Germany) was used.

The evaluation of the modified analytical method included the determination of LOD, LOQ, and the determination of applicable concentration range for *trans*-resveratrol and both ε-viniferin isomers.

All samples were assayed by a validated method and repeatedly measured three times (n = 3). Repeatability was evaluated by testing five repeated injections of standard and sample, while accuracy was evaluated at three different mass concentrations: 0.0816 mg/mL, 0.1632 mg/mL, and 0.3264 mg/mL of standard of resveratrol and 0.1 mg/mL, 0.2 mg/mL, and 0.3 mg/mL of *cis* and *trans*-ε-viniferin solution.

### ^1^H-NMR

The ^1^H NMR spectra were acquired on a Varian INOVA 500 MHz spectrometer (Varian Instruments Inc., USA) operating at a frequency of 499.87 MHz for ^1^H at 298 K. All samples were dissolved in deuterated acetonitrile (ACN-d_3_) and referenced to hexamethyldisilane (HMDSS) internal standard with chemical shift of 0.04 ppm. HMDSS was also used for the *trans*-resveratrol, *cis* and *trans*-ε-viniferin concentration calculation.

### Statistical analysis

Statistical processing of results was performed in SW Excel and The Unscrambler X 10.5.1 (Microsoft, USA). Statistical analysis of results obtained for phenolic compounds included 12 samples of grape pomace and 12 samples of grape cane. The number of samples included in the stability tests was 7. All the samples were measured three times. Averages, standard deviations, and p < 0.01 or p < 0.05 (significant *vs*. non-significant differences), used in statistical analysis were calculated by ANOVA, applying the post hoc Tukey test.

## Conclusion

In this work, the extraction of Cabernet Saugvinon, variety of *Vitis vinifera* L. from Moravian wine region, was extensively studied. Extracts obtained by different extraction methods were exposed to variable stress conditions (light, temperature, and time) in order to evaluate stability of each target compound and easiness of its release from plant matrix. *Trans*-resveratrol, which often remains bound to other molecular structures even in extracts, was found to be relatively thermally stable, but gradually undergoes dimerization when exposed to light.

Extracts containing high amounts of this stilbene can be therefore obtained using extraction in dark at elevated temperatures for time periods no longer than one day. For extraction methods at ambient temperature, longer time periods, such as 14 days, are possible. The main routes of formation of *cis*-ε-viniferin are light-induced dimerization of resveratrol and photoisomerisation of *trans*-ε-viniferin. According to this, the exposition to light during extraction setup will lead to extracts rich in *cis*-ε-viniferin. Elevated temperature should be avoided due to low thermal stability of this stilbene, as well as longer extraction times. To obtain extracts containing high amount of *trans*-ε-viniferin also the protection of material from light during extraction is crucial. Using of elevated temperatures during extraction is in the case of *trans*-ε-viniferin less favorable due to lower thermal stability of this stilbene.

The storage of stilbenes in IPA solution leads to their degradation within 20 days.

## Data Availability

All data generated or analyzed during this study are included in this published article.
